# Special Issue: “Intelligent Systems for Clinical Care and Remote Patient Monitoring”

**DOI:** 10.3390/s23187993

**Published:** 2023-09-20

**Authors:** Giovanna Sannino, Antonio Celesti, Ivanoe De Falco

**Affiliations:** 1 Institute for High-Performance Computing and Networking (ICAR), National Research Council, 80131 Naples, Italy; ivanoe.defalco@icar.cnr.it; 2 Department of Mathematics and Computer Sciences, Physical Sciences and Earth Sciences, University of Messina, 98122 Messina, Italy; acelesti@unime.it

The year 2020 was definitely like no other. COVID-19 shut down the world, and its spread and resulting impact led to a global crisis of unprecedented reach and proportion. This virus has impacted all our lives and forced us to adapt—especially regarding access to healthcare services.

Recent innovations, such as the use of Artificial Intelligence (AI) in healthcare, but also other important technologies, have resulted in better communication and collaboration between medical professionals, improved virtual patient care, and have generated the diffusion of a multitude of medical devices that are saving lives every day. These tools saw an even more massive boost in 2021.

For these reasons, this Special Issue, “Intelligent Systems for Clinical Care and Remote Patient Monitoring”, has focused attention on new technologies, tools, and methodologies for the development of new intelligent systems for clinical care and remote patient monitoring. Among them, we cite micro/nano/cyberphysical systems; AI and machine learning techniques; early pathology detection technologies; e-health, mHealth, telemedicine, and digital solutions; and augmented reality for remote healthcare treatments.

The list of topics related to this Special Issue is quite wide, covering issues related to:

- Fast, cost-effective, and easily deployable sampling, screening, diagnostic, and prognostic systems, including new methods for screening, using, for example, AI, ML, or other advanced solutions;
- Low-cost sensors, smart wearable devices, and robotics/AI for telemedicine, telerehabilitation, telepresence, and continuous remote monitoring of patient parameters;
- Innovative data-driven services, algorithms, and tools for data analysis, data management, and data fusion from various relevant privately held and/or publicly available sources;
- Services for privacy, data protection, and anonymity in the use of mobile healthcare and prevention applications.

The response from the international scientific community has been very positive, and the amount of submitted papers indicates well the large appeal of the topics involved; the selection was very competitive and, in total, 18 manuscripts were accepted.

We are very satisfied with these papers because of, on the one hand, their quality in themselves, and, on the other hand, their illustration of a wide variety of topics discussed, which cover a large part of the issues mentioned in the call for papers.

In the following, we briefly introduce each of them. The presentation of each paper will be based on the authors’ own words so that its contributions can be better presented.

Chew et al. [1] consider the problem of early detection and prevention of cardiovascular diseases by taking into account accessibility for elderly subjects. This is carried out with specific reference to remote and continuous monitoring of vital signs, such as electrocardiograms, and gives rise to the design and deployment of a remote patient monitoring system for arrhythmia detection. This latter consists of a scalable system architecture for the remote streaming of ECG signals in near real-time and is endowed with a two-phase classification scheme. The application of this system to the MIT-BIH Arrhythmia Database shows performance improvement over classical ECG classification algorithms.

Pires et al. [2] present a review of the use of sensors to measure a set of physical parameters to be gathered during the six-minute walk test (6MWT). This test is used to assess aerobic capacity and endurance, thereby allowing for the early detection of emerging medical conditions with changes. This review focuses on various diseases, sensors, and implemented methodologies, and has been carried out by using the PRISMA methodology, which has allowed for the inclusion of 31 papers.

Mezzi et al. [3] focus their research on Arab-speaking subjects and propose an intelligent tool for mental health intent recognition. The aim of this tool is to make diagnoses in mental health, which is accomplished through the combined use of a bidirectional encoder representation from transformers (BERT) model and of the International Neuropsychiatric Interview (MINI). A dataset gathered at the Military Hospital of Tunis is used for the experiments, over which the system shows good performance in the diagnosis of aspects such as depression, suicidality, panic disorder, social phobia, and adjustment disorder. The system has also been used by medical staff and has resulted in interesting and helpful information.

Bernaldo de Quirós et al. [4] present a review of the setups for the measurement of movements of stroke patients under free-living conditions using wearable sensors, and for the evaluation of the relation between such sensor-based outcomes and the level of functioning, as assessed using existing clinical evaluation methods. The review includes 32 articles, and the results contained therein are summarized by type and location of sensors, and by sensor-based outcome measures and their relation to existing clinical evaluation tools. The paper concludes with a call for standardization and consensus.

Na et al. [5] consider human stress in humans and its reduction when human–animal interactions take place, with specific reference to the use of virtual animals. The paper suggests the use of mixed-reality (MR)-based human–animal interaction content and presents the results of its use in reducing stress. To generate mental stress, a mental arithmetic task was proposed to volunteers, after which either interaction with virtual animals or a vision of animal images takes place. For quantitative measurement, an electrocardiogram (ECG) was continuously recorded and their psychological state was evaluated with the help of questionnaires after each task. The MR-based interaction with virtual animals has turned out to reduce mental stress significantly.

Senk et al. [6] deal with Tactile Internet (TI) with human-in-the-loop, an improvement in tele-healthcare that is expected to positively impact care provisioning. One of the TI challenges lies in the need for ultra-low latency. Within this framework, the authors list the benefits that could be obtained through the solution of the network latency reduction challenge; they also note that this could be achieved through the use of Time-Sensitive Networking (TSN) devices. This would give rise to a set of new services that could be obtained; they would positively impact issues such as remote surgery, remote rehabilitation, and tele-healthcare in rural settings.

Makroum et al. [7] perform a systematic review of articles describing the use of smart devices for the management of diabetes. This review includes 89 papers, dating from 2011 to 2021, in which the use of wearable devices takes place in conjunction with Machine Learning methodologies to perform several tasks typical of diabetes management, such as the prediction of future blood glucose values, early detection of dangerous situations such as hypo- or hyper-glycemic events, and the automatic adjustment of insulin doses. These methodologies have turned out to be very helpful in the management of diabetes and in the improvement of life quality.

Chimamiwa et al. [8] take into account elderly people with dementia and consider that smart homes, although extremely useful in terms of activity recognition and anomaly detection, cannot gather information on how subjects’ habits change over time as long as dementia advances. This goal would need the recognition of habits and of their changes as well. In the paper, the authors provide an overview of the stages of dementia, perform a survey on the topic, classify the relevant literature, and discuss the challenges of the implementation of habit recognition in smart homes for elderly people with dementia.

Mavrogiorgou et al. [9] aim to evaluate the efficiency of a set of widely used Machine Learning algorithms to face several healthcare problems where timely decision-making should be performed. To accomplish this, they consider seven such Machine Learning algorithms (Naïve Bayes, K-Nearest Neighbors, Decision Tree, Logistic Regression, Random Forest, Neural Networks, Stochastic Gradient Descent) and six important healthcare scenarios, namely stroke, COVID-19, diabetes, breast cancer, kidney disease, and heart failure. They find that, for any considered scenario, a subset of the considered algorithms results in more efficient outcomes.

Prokopowicz et al. [10] propose a hierarchically structured fuzzy model to quantify satisfaction in Quality of Life and to detect changes in its score values. Their model relies on a set of four clinometric scales related to stress, burnout, satisfaction with life, and musculoskeletal status; these scales are combined through fuzzy solutions. To obtain data useful for the experiments, two groups of specialists at risk of occupational burnout were assessed three times at different intervals in terms of life satisfaction. The authors claim that their approach is novel because, for any individual, three consecutive time points are considered and because fuzzy logic is used.

Ubl et al. [11] face the problem of the evaluation of controllers for insulin pumps to be used by patients with diabetes to correctly dose insulin; this is an extremely important task for safety reasons. They propose an evaluation method that takes advantage of a diabetic patient simulator approved by the FDA. The method evaluates the cartesian product of individual insulin pump parameters with a fine degree of granularity, hence allowing the identification of both safe and risky combinations of insulin pump parameter settings, which helps assess controller safety. The approach is tested over two existing controllers, Low-Glucose Suspend and OpenAPS, and allows the discovery of the best settings and of good setting regions among them.

Ferraris et al. [12] describe the REHOME project, whose main goal consists of meeting the needs of both clinicians and patients in ensuring continuity of treatment, from healthcare facilities to the patient’s home. In particular, elderly or pathological subjects with neurological diseases are considered: the solution usability should be ensured to them. The project is based on technological solutions integrating innovative methodologies and devices for remote monitoring and rehabilitation of cognitive, motor, and sleep disorders associated with neurological diseases. The paper also discusses the results from a set of questionnaires on usability and user experience completed by people participating in the experimentation.

Luo et al. [13] face the problem of timely diagnosis and treatment of retinopathy in remote areas where medical resources are scarce. To this aim, they propose a telemedicine system that is based on a collaborative edge-cloud architecture and is endowed with a deep neural network algorithm carrying out the task of classifying the pre-processed eye images. This classification algorithm is based on ResNet101, uses undersampling and resampling to efficiently tackle the problem of data imbalance, and is embedded in mobile devices.

Silvestri et al. [14] consider the problem of understanding and addressing cyber threats in healthcare systems; these can come both from the connected medical devices and from other parts of the ICT health infrastructure. The authors propose an effective way to analyze threats and vulnerabilities that is based on the use of Machine Learning models, namely the BERT neural language model and XGBoost. The proposed approach allows one to extract updated information from the Natural Language documents widely available on the Web, evaluating at the same time both the level of the identified threats and the vulnerabilities that can impact the healthcare system, and providing the information that is necessary to manage the risk in the most appropriate way.

Torres-Guzman et al. [15] discuss the problems of remote fall detection and prevention for elderly people. They perform a review on the use of smartphones that lists 44 papers of interest. The vast majority of them just consider fall detection, and only three of them consider fall prevention. Among the results of this review, the authors highlight that many papers perform analysis on data coming from an accelerometer, use data related to previous falls, and avail themselves of machine learning methodologies as possible ways to improve accuracy in the detection of falls. Furthermore, a relevant conclusion is that smartphones seem promising for fall detection, but their use is still not wide.

Manouchehri et al. [16] investigate the problem of human activity recognition, which is important in various eHealth domains. For the analysis of human activities, they consider that these have sequential patterns and that hidden Markov Models are very effective at modeling data with continuous flow. Consequently, they propose unsupervised, scaled, Dirichlet-based hidden Markov models. The assumption is made that the hidden Markov Model’s emission probabilities are distributed according to a bounded-scaled Dirichlet distribution. Furthermore, in the learning phase, a variational inference approach is used.

Brancato et al. [17] investigate breast cancer prognosis; to improve its accuracy, they introduce a new radiomic approach that can build predictors for a set of four markers that are known to be important for this prognosis task. These markers are the Estrogen Receptor, Progesterone Receptor, Human Epidermal Growth Factor Receptor 2, and Ki67 antigen. The approach they propose is based on a two-step feature selection process that allows one to build predictors for these four markers. The use of an in-house dataset allowed them to obtain very good results for the four markers in terms of F1-score.

Hassan et al. [18] consider retinal optical coherence tomography (OCT) imaging, which is very important in assessing the status of the back of the eye with respect to both primary eye diseases and systemic ones, e.g., those related to diabetes. Within this field, they propose an automated Deep Learning procedure relying on an enhanced OCT model for the classification of retinal OCTs. This model is based on the use, during the training phase, of modified ResNet and of Random Forest algorithms. Adam optimizer is also utilized during training. This results in very good numerical values for the typical parameters used in the evaluation of classification quality.

We cannot conclude this Editorial without expressing our most sincere gratitude to all the main actors of the present Special Issue, whose roles have been highly important for its favorable outcome.

Firstly, we wish to show appreciation to the authors for contributing their innovative views and proposals and for choosing this Special Issue to introduce their ideas to the worldwide scientific community.

In addition to this, we wish to thank the reviewers for their hard work in assessing the quality of the submissions as well as in supplying the authors with well-founded proposals and recommendations aiming to further improve the submitted manuscripts.

Very importantly, despite being mentioned at the end of this list, we wish to wholeheartedly thank everyone at *Sensors*: their constant support in the management of this Special Issue was exceedingly helpful and made our Editorship straightforward.

The synergy of the combined efforts from all of the above participants has made the publication of this Special Issue in *Sensors* possible; it contains, we believe, novel, stimulating, and pertinent contributions to “Intelligent Systems for Clinical Care and Remote Patient Monitoring”. We believe that these contributions can constitute valid foundations that could be used worldwide by the scientific community to carry out new research in the future.

## Data Availability

All data have been presented in the main text.
